# Correction: Large-scale population disappearances and cycling in the white-lipped peccary, a tropical forest mammal

**DOI:** 10.1371/journal.pone.0314917

**Published:** 2024-12-02

**Authors:** José M. V. Fragoso, André P. Antunes, Kirsten M. Silvius, Pedro A. L. Constantino, Galo Zapata-Ríos, Hani R. El Bizri, Richard E. Bodmer, Micaela Camino, Benoit de Thoisy, Robert B. Wallace, Thais Q. Morcatty, Pedro Mayor, Cecile Richard-Hansen, Mathew T. Hallett, Rafael A. Reyna-Hurtado, H. Harald Beck, Soledad de Bustos, Alexine Keuroghlian, Alessandra Nava, Olga L. Montenegro, Ennio Painkow Neto, Mariana Altrichter

The fourteenth author’s name is spelled incorrectly. The correct name is: Matthew T. Hallett.

## References

[1] Fragoso JMV, Antunes AP, Silvius KM, Constantino PAL, Zapata-Ríos G, Bizri HRE, et al. (2022) Large-scale population disappearances and cycling in the white-lipped peccary, a tropical forest mammal. PLOS ONE 17(10): e0276297. 10.1371/journal.pone.0276297 36264921 PMC9584423

