# Supplementary material for: Large-scale population disappearances and cycling in the white-lipped peccary, a tropical forest mammal
Source: PLoS One. 2022 Oct 20;17(10):e0276297. doi: 10.1371/journal.pone.0276297 (PMC9584423; doi:10.1371/journal.pone.0276297)
Supplement: S1 Table — (DOCX) [file pone.0276297.s001.docx]

S1: Questions emailed to researchers that work with white-lipped peccaries (*Tayassu pecari*).

1. Your name, institution, research topic.
2. Have you or any one you know observed unexplained disappearances of white-lipped peccary populations?
3. If yes, who noted or recorded the disappearance? Name of observer and contact information.
4. What was the country and site/s location (longitude and latitude) where the disappearance occurred?

For those that responded affirmatively we asked the subsequent questions:

1. What was the date or approximate date the WLP disappearance?
2. What date or approximated date did the WLP re-appear?
3. Was there more than one disappearance event in this area or nearby?
4. If yes to 3, what was the location and date of previous or subsequent disappearances and re-appearances?
5. Please give the size of the area from which WLP disappeared; please estimate area size to the best of your ability.
6. Were any WLP carcasses observed when a disappearance occurred?
7. If carcasses were observed, could the cause of death be identified?
8. If yes to 7, what was the nature of the evidence?
9. Did you or anyone else publish a record of said disappearance event?
10. If yes to 9, please give a reference for the publication.
11. What method did you and your colleagues use when monitoring WLP populations for your observation?
