# Supplementary material for: Large-scale population disappearances and cycling in the white-lipped peccary, a tropical forest mammal
Source: PLoS One. 2022 Oct 20;17(10):e0276297. doi: 10.1371/journal.pone.0276297 (PMC9584423; doi:10.1371/journal.pone.0276297)
Supplement: S2 Table — (DOCX) [file pone.0276297.s002.docx]

S2: Reports from experts on 43 independent white-lipped peccaries (*Tayassu pecari*) disappearances at 38 sites in 9 countries.

| **Country** | **Reporter** | **Observers** | **Region** | **Area** | **Approximate area km^2^** | **Latitude** | **Longitude** | **Date(s) disappeared** | **Date(s) re-appeared** | **Years absent** | **Methods** | **Source** |
| --- | --- | --- | --- | --- | --- | --- | --- | --- | --- | --- | --- | --- |
| Argentina | Camino M | Di Bitetti M, Varela D, Cruz P, Paviolo A, JP Arrabal JP | Atlantic Forest | Iguazú National Park and surroundings | 7 133 | 25° 38' S | 54° 17' W | 1995 - 2000 | 2016-2019 | 19-21 | Camera traps | [1], Di Bitetti M, Varela D, Cruz P, Paviolo A, Arrabal JP, unpub. data |
| Argentina | de Bustos S | de Bustos S, Garay D, Perovic P, Reppucci J, Maras G, Alveira M, Bardavid S, Rivera L, Politi N. | Yungas (Mountain Forest), Salta | Serranías de Tartagal | 1 540 | 22° 26' S | 63° 59' W | 2000 - 2005 | Still absent | Still absent | Line transects; camera traps | [2-6] |
| Argentina | de Bustos S | de Bustos S, Falke F, Correa M, Perovic P,  Reppucci J, Maras G, Bardavid S, Rivera L, Politi N. | Yungas (Mountain Forest) in Salta, Argentina | Baritú - Pintascayo Region | 2 640 | 22° 50' S | 64° 40' W | 2008-2010 | Still absent | Still absent | Line transects; camera traps | [3-6] |
| Argentina | de Bustos S | de Bustos S, Perovic P, Reppucci J, Maras G, Bardavid S, Rivera L, Politi N, Vallejos J. | Yungas (Mountain Forest) in Jujuy and Salta, Argentina | Calilegua National Park and surroundings | 1 050 | 23° 31' S | 64° 37' W | 2010 - 2015 | Still absent | Still absent | Line transects; camera traps | [3-7], Grassman L, Rocca F, unpub. data |
| Bolivia | Fragoso JMV | Stearman AM | Santa Cruz and Cochabamba Departments | Yuqui Indigenous Area | 1 150 | 16° 47' S | 64° 56' W | 1985 | 1995 | 10 | Literature | [8, 9] |
| Bolivia | Wallace R | Wallace R | Madidi National Park | Heath River | 400 | 13° 0' S | 68° 50' W | 2017 | Still absent | Still absent | Camera trapping, interviews | Wallace R, unpub. data |
| Bolivia | Wallace R | Ayala G, Wallace R | Madidi National Park | Tuichi valley and Hondo valley | 400 | 14° 39' S | 67° 42' W | (a) 1984 - 1989 / (b) 2018 | (a) 1999 / (b) still absent 2020 | (a) 15 / (b) Still absent | Camera trapping, interviews | Wallace R, unpub. data |
| Bolivia | Wallace R | Gomez H, Gottdenker N | Madidi National Park | Tuichi valley | 400 | 14° 39' S | 67° 42' W | (a) Mid 1990's / (b) 2018 | (a) 1999 / (b) Still absent-2020 | (a) ~5 / (b) Still absent | Occasional encounters (dead individuals) | Wallace R, unpub. data |
| Bolivia | Wallace R | Several local guides | Madidi National Park | Tuichi valley and Hondo valley | 400 | 14° 30' S | 67° 40' W | 2018 | Still absent-2020 | Still absent | Interviews, observations, occupancy, camera trapping | Wallace R, unpub. data |
| Bolivia | Wallace R | The Tacana people | Northern La Paz Department | Madidi National Park | 10 000 | 14° 7' S | 67° 29' W | Historical | Historical | Still absent | Interviews, indigenous oral folklore | Cardenas C, Lara K, unpub. data |
| Bolivia | Wallace R | Two local hunters | Northern La Paz Department | Ixiamas municipallity | 1 000 | 14° 25' S | 67° 42' W | Early 1990's | Late 1990´s | 8 | Occasional encounters (dead individuals) | WCS-Bolivia, unpub. data |
| Brazil | Nava A | Nava A | São Paulo State | Morro do Diabo State Park | 338 | 22° 31' S | 52° 17' W | 2011 | Still absent | Still absent | Line transects | Nava A, unpub. data |
| Brazil | Keuroghlian A, Dezbiez A | Chico Mendes Institute for Biodiversity Conservation (ICMBio) | Rio Grande do Sul State | Turvo State Park | 175 | 27° 11' S | 53° 53' W | 1980 | Still absent | Still absent | Literature | [10-12] |
| Brazil | Keuroghlian A, Dezbiez A | Chico Mendes Institute for Biodiversity Conservation (ICMBio) | São Paulo State | Intervales State Park | 417 | 24° 18' S | 48° 16' W | 1990 | 2017 | 25 | Camera trapping | [11-13] |
| Brazil | Keuroghlian A, Dezbiez A | Chico Mendes Institute for Biodiversity Conservation (ICMBio) | São Paulo State | Carlos Botelho State Park | 380 | 24° 9' S | 48° 3' W | 1997 | 2012 | 15 | Camera trapping | [13,14] |
| Brazil | Keuroghlian A, Dezbiez A | Chico Mendes Institute for Biodiversity Conservation (ICMBio) | São Paulo State | Morro do Diabo State Park | 338 | 22° 31' S | 52° 17' W | 2010 | 2016 | 6 | Literature | [11], Cullen C, unpub. data |
| Brazil | Painkaw E | Painkaw E | Pará State | Saracá-Taquera National Forest | 4 413 | 1° 38' S | 56° 27' W | 2017 | Still absent | Still absent | Line transects, camera trapping | Painkow E, unpub. data |
| Brazil | El Bizri H | Valsecchi J | Amazonas State | Auatí-Paraná Extractive Reserve | 1 470 | 2° 23' S | 66° 40' W | 2011 | Still absent | Still absent | Interviews | Valsecchi J; unpub. data |
| Brazil | El Bizri H | El Bizri H, Valsecchi J | Amazonas State | Amanã Sustainable Development Reserve | 23 500 | 2° 33' S | 64° 43' W | 2012 - 2014 | Still absent | Still absent | Camera trapping, hunting records, interviews | [15] |
| Brazil | Fragoso JMV | Fragoso JMV | Roraima State | Yanomami Indigenous Area, Maracá Island Ecological Reserve, and surrounding forests | 56 074 | 3° 22' N | 61° 28' W | 1989 | 2005 | 16 | Line transects, radio tracking, interviews | [16-18] |
| Brazil | Fragoso JMV | Borges LHM | Acre State | Chandless State Park | 6 953 | 9° 44' S | 70° 30' W | 2006 | Unknown | Unknown | Line transects, camera trapping, interviews | [19] |
| Brazil | Fragoso JMV | Fragoso JMV | Pará State and Amapá State | Tumucumaque Indigenous Park and East Paru Indigenous Park | 42 669 | 1° 16' N | 55° 37' W | 2012 | Unknown | Unknown | Line transects, hunting records | Fragoso JMV, unpub. data |
| Brazil | Fragoso JMV | Fragoso JMV | Pará State, Amazonas State and Roraima State | Trombetas/Mapuera Indigenous Land and Nhamundá-Mapuera Indigenous Land | 50 199 | 0° 17' S | 58° 3' W | 2012 (Uncertain) | Unknown | Unknown | Line transects, hunting records | Fragoso JMV, unpub. data |
| Brazil | Fragoso JMV | Sr. Santos Jr. | Amazonas State | Unini Extractive Reserve | 8 334 | 1° 43' S | 63° 0' W | 2012 | Still absent | Still absent | Line transects, interviews | Santos Sr, unpub. data |
| Brazil | Fragoso JMV | Fragoso JMV | Roraima State | Viruá National Park | 2 420 | 1° 17' N | 61° 7' W | 2016 | Still absent | Still absent | Occasoinal encounters (ill individuals) | Fragoso JMV, unpub. data |
| Brazil | Mangini P | Crawshaw P, Mangini P, Brocardo CR | Paraná State | Foz do Iguaçu National Park | 1 853 | 25° 40' S | 54° 27' W | 1995 | 2016 | 21 | Line transects, camera trapping | [1, 20], Crawshaw P, Mangini P, unpub. data. |
| Brazil | Constatino P | Constatino P | Acre State and Amazonas State | Upper Juruá and Purus Rivers, Brazil-Peru frontier | 30 915 | 8° 46' S | 72° 7' W | 2010 | 2017 | 7 | Hunting records | Constatino P unpub. data |
| Brazil | Morcatty T | Morcatty T | Amazonas State | Jutaí River Extractive Reserve | 2 755 | 3° 18' S | 67° 15' W | (a) 1998 / (b) 2002 / (c) 2004 | (a) 2006 / (b) 2006 / (c) 2011 | (a) 8 / (b) 6 / (c) 7 | Interviews | Morcatty T, unpub. data |
| Brazil | Morcatty T | Morcatty T | Amazonas State | Unini Extractive Reserve | 8 334 | 1° 43' S | 63° 0' W | 2013 | Still absent | Still absent | Interviews | Morcatty T, unpub. data |
| Brazil | Morcatty T | Morcatty T | Amazonas State | Jaú National Park | 23 673 | 1° 46' S | 62° 13' W | 1993 | 1999 | 5 | Interviews | Morcatty T, unpub. data |
| Brazil | Morcatty T | Morcatty T | Amazonas State | Jaú National Park | 23 673 | 2° 2' S | 62° 5' W | 2005 | 2009 | 4 | Interviews | Morcatty T, unpub. data |
| Brazil | Morcatty T | Morcatty T | Amazonas State | Jaú National Park | 23 673 | 1° 49' S | 62° 49' W | 2009, 2012, 2014 | Still absent | Still absent | Interviews | Morcatty T, unpub. data |
| Brazil | Morcatty T | Morcatty T | Amazonas State | Jutaí River Extractive Reserve | 2 755 | 3° 3' S | 67° 6' W | 2013 - 2015 | Still absent | Still absent | Interviews | Morcatty T, unpub. data |
| Brazil, Bolivia, Peru | Antunes A | Antunes A | Rondônia State | Porto Velho, upper Madeira River | Uncertain | 8°45′ S | 63°54′ W | 1945 | 1952 | 7 | Hunting records | Antunes A, unpub. data |
| Brazil, Bolivia, Peru | Antunes A | Antunes A | Amazonas State | Boca do Acre, Acre River, upper Purus River | Uncertain | 8°45' S | 67°23′ W | 1945? | Unknown | Unknown | Hunting records | Antunes A, unpub. data |
| Brazil, Bolivia, Peru | Antunes A | Antunes A | Acre State | Rivaliza, Cruzeiro do Sul, upper Juruá River | Uncertain | 7°37′ S | 72°40′ W | 1946 | ~1959 | ~13 | Hunting records | Antunes A, unpub. data |
| Brazil, Bolivia, Peru | Antunes A | Antunes A | Amazonas State | Seringal Macapá, upper Purus River | Uncertain | 8°45′ S | 67°23′ W | 1949 | 1959 | 10 | Hunting records | Antunes A, unpub. data |
| Brazil, Bolivia, Peru | Antunes A | Antunes A | Acre State and Amazonas State | Riozinho da Liberdade, upper Juruá River | 5 626 | 7° 42' S | 72° 0' W | 1949 | 1953 | 4 | Hunting records | Antunes A, unpub. data |
| Brazil, Bolivia, Peru | Antunes A | Antunes A | Acre State | Iaco River, upper Purus River | 24 837 | 8° 41' S | 71° 40' W | 1949 | 1961 | 12 | Hunting records | Antunes A, unpub. data |
| Colombia | Montenegro O | Montenegro O, von Hildebrand P | Chiribiquete National Park | Southern Chiribiquete National Park | 12 990 | 0° 0' N | 72° 21' W | 1990 | ~2000 | ~10 | Line transects | Montenegro O, unpub. data |
| Ecuador | Zapata-Rios G | The Shuar people | Morona-Santiago Province | Miasal Shuar Indigenous Area | 2 000 | 2° 37' S | 77° 47' W | 2000 | 2007 | 7 | Line transects, hunting records, interviews | Zapata-Rios G, unpub data |
| Ecuador | Fragoso JMV | Vickers WT | Siona-Secoya Inidgenous Area | Siona-Secoya Indigenous Area | 27 824 | 0° 16' S | 76° 1' W | 1975 | 1985 | ~10 | Literature | [21] |
| French Guiana | de Thoisy B | de Thoisy B | Entire state | Entire state | 80 000 | 3° 35' N | 53° 9' W | 2003 | 2012 | ~9 | Line transects | de Thoisy B, unpub. data |
| French Guiana | Richard-Hansen C | Richard-Hansen C | Entire state | Entire state | 80 000 | 3° 35' N | 53° 9' W | 2007 - 2008 | 2012 - 2013 | ~7 | Line transects, hunting records | [22], Richard-Hansen C, unpub data |
| Guatemala | McNab R | McNab R | Maya Biosphere Reserve | Laguna del Tigre National Park | 500 | 17°25′ N | 90° 53′ W | 2016 | Unknown | Unknown | Camera trapping | McNab R, unpub. data |
| Guyana | Fragoso JMV | Major General J. Singh (Protected Areas Trust Guyana) | Region 9 | Konashen (Wai Wai Community Reserve) | 6 070 | 1° 45' N | 58° 56' W | 2012 | Still absent | Still absent | Interviews | Singh J, Protected Areas Trust, unpub. data |
| Guyana | Fragoso JMV | Overman H | Region 8 | North Rupununi | 48 000 | 1° 31' N | 58° 47' W | 2011-12 | Still absent | Still absent | Line transects | North Rupununi Development Board, unpub. data |
| Guyana | Hallett M | Hallett M | Region 9 | Kanuku Mountains | 6 110 | 3° 16' N | 59° 22' W | ~2011 | Still absent | Still absent | Camera trapping | [23] |
| Guyana | Hallett M | Hallett M | Region 9 | Kanuku Mountains | 6 110 | 3° 16' N | 59° 22' W | ~2011 | Still absent | Still absent | Camera trapping | [23] |
| Guyana | Hallett M | Hallett M | Regions 8 and 9 | Iwokrama Centre for Rainforest Conservation & Development - Sustainable Utilization Area | 3 717 | 3° 16' N | 59° 22' W | ~2011 | 2015 - current | ~4 | Camera trapping | [23] |
| Guyana | Hallett M | Hallett M | Regions 8 and 9 | Iwokrama Centre for Rainforest Conservation & Development - Wilderness Area | 3 717 | 4° 35' N | 58° 43' W | ~2011 | 2015 | ~4 | Camera trapping | [23] |
| Guyana | Hallett M | Hallett M | Region 9 | Head of the Rewa River | 300 | 2° 45' N | 58° 37' W | ~2011 | 2015 | ~4 | Camera trapping | [23] |
| Guyana | Hallett M | Hallett M | Region 9 | Rewa village area | 300 | 3° 58' N | 58° 46' W | ~2011 | 2016 - current | ~5 | Camera trapping | [23] |
| Guyana | Hallett M | Hallett M | Region 9 | Surama village area | 60 | 4° 10' N | 59° 5' W | ~2011 | 2015 - 2016 | ~5 | Camera trapping | [23] |
| Guyana | Hallett M | Hallett M | Region 9 | North Rupununi wetlands | 1 200 | 1° 31' N | 58° 47' W | ~2011 | Still absent | Still absent | Camera trapping | [23] |
| Guyana | Hallett M | Hallett M | Region 9 | Southern Kanuku Mountains and South Rupununi savannas | 1 500 | 2° 49' N | 59° 31' W | ~2011 | Still absent | Still absent | Camera trapping | [23] |
| Guyana | Hallett M | Hallett M | Region 9 | Kanuku Mountain communities | 6 111 | 3° 16' N | 59° 22' W | ~2011 | Still absent | Still absent | Interviews | [23] |
| Guyana | Hallett M | Hallett M | Region 9 | Saddle Mountain Ranch | 150 | 2° 48' N | 59° 49' W | Never | Maintained to current | 0 | Camera trapping | [24] |
| Guyana | Hallett M | James T | Region 9 | Aishalton village area | 564 | 2° 30' N | 59° 15' W | ~2011 | 2016 | ~5 | Interviews | James T, unpub. data |
| Guyana | Fragoso JMV | Fragoso JMV | Regions 8 and 9 | Rupununi and Karasabai Regions | 48 000 | 1° 31' N | 58° 47' W | 2011 | Still absent | Still absent | Line transects, camera trap, interviews | [23, 25-27] |
| Peru | Emmons L | Emmons L | Madre de Dios Department and Cusco Department | Manu National Park | 71 000 | 11° 53' S | 71° 24' W | 1979 | 1992 |  | Line transects, interviews | [28-31] |
| Peru | Mayor P | Mayor P | Rural communities Boca Manu and Islas de los Valles | Manu National Park buffer zone | 100 | 12° 15' S | 70° 15' W | 1983 | 1998 | 15 | Interviews | Mayor P, unpub. data |
| Peru | Mayor P | Mayor P | Rural community Shintuya | Manu National Park buffer zone | 100 | 12° 41' S | 71° 15' W | 1983 | 1998 | 15 | Interviews | Mayor P, unpub. data |
| Peru | Mayor P | Mayor P | Yavarí-Mirín River | Nueva Esperanza indigenous village communal area (Yagua People) | 1 923 | 4° 48' S | 71° 78′ W | 2004 | 2016 |  | Line transects, hunting records, interviews | [32] |
| Peru | Wallace R | Ayala G, Viscarra M | Manu National Park | Manu River and Panagua River | 300 | 12° 2' S | 71° 43' W | 2013 | Still absent | Still absent | Camera trapping, interviews | WCS-Peru, unpub. data |
| Peru | Wallace R | Carlos N, Brightsmith D | Tambopata National Reserve | Tambopata Research Center | 100 | 13° 8' S | 69° 36' W | 2013 - 2014 | Still absent | Still absent | Occasional encounters (dead individuals) | Carlos N, Brightsmith D, unpub. data |
| Peru | Beck H | Terborgh J, Alvares P, Beck H | Madre de Dios Department and Cusco Department | Manu National Park | 17 163 | 11° 53' S | 71° 24' W | 2011 - 2014 | 2015 | ~2-3 | Camera trapping | Terborgh J, unpub. data |
| Peru | Fragoso JMV | Silman K, Terborgh J | Madre de Dios Department and Cusco Department | Manu National Park | 17 163 | 11° 53' S | 71° 24' W | 1978 | 1990 |  | Literature | [31] |
| SW Amazon Biome | Antunes A, Constantino P | Antunes A, Constantino P | SW Amazon Basin, SE Peru, SW Brazil and NW Bolivia |  | 6 860 000 | 9° S | 70° W | (a) 1950 / (b) 1985 / (c) 2010 | (a) 1965 / (b) 1995 / (c) still absent | (a) 15 / (b) 10 / (c) still absent | Hunting records | Antunes A, Constantino P, unpub. data |

1. Azevedo FCC, Conforti VA. Decline of peccaries in a protected subtropical forest of Brazil: toward conservation issues. Mammalia 2008; 2: 82-88.

2. Barbarán F. Estado del hábitat y registros de la presencia del tigre (*Panthera onca*) en el área de influencia de la Reserva Provincial Acambuco (Provincia de Salta, Argentina). Ecosistemas 2004;13: 88-95.

3. Taber A, Chalukian SC, Minkowki K, Lizzaraga L, Sanderson EW, Rumiz D, et al. El destino de los arquitectos de los bosques neotropicales: evaluación de la distribución y estado de conservación de los pecaríes labiados y los tapires de tierras bajas. IUCN/SSC Tapir Specialist Group and Peccary Specialist Group, IUCN, Wildlife Conservation Society & Wildlife Trust; 2008.

4. de Bustos S. Situación crítica del pecarí labiado *Tayassu pecari* en las Yungas de la Alta Cuenca del Río Bermejo, Argentina. In Libro de Resumen XXII Jornadas Argentinas de Mastozoología, Argentina, Sociedad Argentina para el Estudio de los Mamíferos; 2018.

5. Bardavid S, Bustos S, Politi N, Rivera L. Escasez de registros de pecarí labiado (*Tayassu pecari*) en un sector de alto valor de conservación de las Yungas australes de Argentina. Mastozool. Neotrop*.* 2019;26: 167-173.

6. de Bustos S, Varela D, Lizárraga L, Camino M, Quiroga VA*. Tayassu pecari*. Categorización 2019 de los mamíferos de Argentina según su riesgo de extinción. Lista Roja de los mamíferos de Argentina (SAyDS–SAREM eds.). 2019.

7. Perovic P. Ecología de la comunidad de félidos en las Selvas Nubladas del Noroeste Argentino. Ph.D. dissertation. Universidad Nacional de Córdoba. 2002.

8. Stearman AM. Making a living in the tropical forest: Yuqui foragers in the Bolivian Amazon. Hum. Ecol. 1991;19: 245-260.

9. Stearman AM, Redford KH. Game management and cultural survival: the Yuqui ethno development project in lowland Bolivia. Oryx 1995;29: 29-34.

10. Wallauer JP, Albuquerque EP. Lista preliminar dos mamíferos de observados no Parque Estadual do Turvo, Tenente Portela, Rio Grande do Sul, Brasil. Roessléria 1986;**8**: 179-185.

11. Keuroghlian A, Desbiez ALJ, Beisiegel B Medici EP, Gatti A, Pontes A, et al. Avaliação do risco de extinção do queixada, *Tayassu pecari* (Link, 1795) no Brasil*.*BioBrasil. 2012;3: 84-102.

12. Kasper CB, Mazim FD, Soares JBG, Oliveira TG, Fabian ME. Composição e abundância relativa dos mamíferos de médio e Grande Porte no Parque Estadual do Turvo, Rio Grande do Sul, Brasil. Rev. Bras. Zool. 2007;24: 1087-1100.

13. Beisiegel BM, Nakano E, Jorge MLSP. Are white-lipped peccaries back in the Paranapiacaba Forest, São Paulo, Brazil? Suiform Soundings 2014;12: 29-33.

14. Beisiegel BM. Shelter availability and use by mammals and birds in an Atlantic forest area. Biota Neotropica 2006;6: 1-16.

15. Rocha DG. Padrão de atividade e fatores que afetam a amostragem de mamiferos de Medio e Grande Porte na Amazonia. MSc. dissertation. Instituto Nacional de Pesquisas da
Amazônia Central. 2015.

16. Fragoso JMV. Desapariciones locales del baquiro labiado (*Tayassu pecari*) en la Amazonía: migración, sobre-cosecha o epidemia. In Fang T, Bodmer R, Aquino R, Valqui M. editors. Manejo de Fauna Silvestre en la Amazonía*.* United Nations Development Program-Global Environmental Facility, Universidad de Florida, Instituto de Ecología. La Paz, Bolivia; 1997. pp. 309-312.

17. Fragoso JMV. A long-term study of white-lipped peccary (*Tayassu pecari*) population fluctuation in northern Amazonia. In Silvius K, Bodmer RE, Fragoso JMV, editors. People in Nature, Wildlife Conservation in South and Central America*.* Columbia University Press, New York; 2004. pp. 286-296.

18. Fragoso JMV. Large mammals and the community dynamics of an amazonian forest. Ph.D. Dissertation. University of Florida. 1994.

19. Borges LHM, Calouro AM, Sousa JR Large and medium-sized mammals from Chandless State Park, Acre, Brazil*.* Mastozool. Neotrop*.* 2015;22: 265-277.

20. Brocardo CR, da Silva MX, Delgado LE, Galetti M. White-lipped peccaries are recorded at Iguaçu National Park after 20 years. Mammalia 2017;81: 519-522.

21. Vickers WT. Hunting yields and game composition over ten years in an Amazon Indian territory. Neotropical Wildlife Use and Conservation 1991;400: 53-81.

22. Richard-Hansen C, Surugue N, Khazraie K, Le Noc M, Grenand P. Long-term fluctuations of white-lipped peccary populations in French Guiana. Mammalia 2014;78 291-301.

23. Hallett MT, Kinahan AA, McGregor R, Baggallay T, Babb T, Barnabus H, et al. Impact of low-intensity hunting on game species in and around the Kanuku Mountains Protected Area, Guyana. Front. Ecol. Evol. 2019;7: 412.

24. Hallett MT. Landscape-scale research as a tool for engaging communities in a shared learning process for conservation and management in the Rupunuini, Guyana, Ph.D. Dissertation, University of Florida. 2017.

25. Paemelaere EAD, Fernandes D, Leroy I, Angelbert J. Large mammals of the South Rupununi Region, Guyana. Biodiversity assessment survey of the South Rupununi Savannah, Guyana. Alonso LE, Persaud J, Williams A, editors. BAT Survey Report No. 1. WWF-Guianas, Guyana Office. Georgetown, Guyana; 2016.

26. Roopsind A, Caughlin TT, Sambhu, Fragoso JMV, Putz FE. Logging and indigenous hunting impacts on persistence of large Neotropical animals. Biotropica 2017;49: 565-575.

27. Shaffer CA, Milstein MS, Yukuma C, Marawanaru, E, Suse P. Sustainability and comanagement of subsistence hunting in an indigenous reserve in Guyana. Biol. Conserv*.* 2017;**31**: 1119-1131.

28. Kiltie KA Distribution of palm fruits on a rain forest floor: why white-lipped peccaries forage near objects. Biotropica 1981;14: 141-145.

29. Emmons LH, Comparative feeding ecology of felids in a neotropical rainforest*.*Behav. Ecol. Sociobiol. 1987;20: 271-283.

30. Kiltie RA, Terborgh J. Observations on the behavior of rain forest peccaries in Peru: Why do white-lipped peccaries form herds? *Z. Tierpsychol.* 1983;62: 241-255.

31. Silman MR, Terborgh J, Kiltie R. Population regulation of a dominant-rain forest tree by a major seed-predator. Ecology 2003;84: 431-438.

32. Mayor P, Pérez-Peña P, Bowler M, Puertas PE, Kirkland M, Bodmer P. Effects of selective logging on large mammal populations in a remote indigenous territory in the northern Peruvian Amazon. Ecol. Soc*.* 2015; 20: 36.
