# Supplementary material for: Large-scale population disappearances and cycling in the white-lipped peccary, a tropical forest mammal
Source: PLoS One. 2022 Oct 20;17(10):e0276297. doi: 10.1371/journal.pone.0276297 (PMC9584423; doi:10.1371/journal.pone.0276297)
Supplement: Attachment — Submitted filename: PONE-D-21-25351_R.pdf [file pone.0276297.s003.pdf]

PLOS ONE

Disappearances and population cycles of a large mammal in Neotropical forests

--Manuscript Draft--

Manuscript Number:	PONE-D-21-25351
Article Type:	Research Article
Full Title:	Disappearances and population cycles of a large mammal in Neotropical forests
Short Title:	Mysterious Peccary Dissapearances
Corresponding Author:	Jose M.V. Fragoso<br>California Academy of Sciences<br>San Francisco, CA UNITED STATES
Keywords:	Population cycle; density dependence; faunal collapse; defaunation; fragmentation
Abstract:	Many vertebrate species undergo population fluctuations that may be random or regularly cyclic in nature. Vertebrate population cycles in northern latitudes are driven by both endogenous and exogenous factors. Suggested causes of mysterious disappearances documented for populations of the Neotropical, herd-forming white-lipped peccary (Tayassu pecari , henceforth “WLP”) include long distance or migratory movements, overhunting, extreme floods, or disease outbreaks. By analyzing 43 disappearance events across the Neotropics and 88 years of commercial and subsistence harvest data for the Amazon, we show that WLP disappearances are widespread and occur regularly and at large spatiotemporal scales throughout the species’ range. We present evidence that the disappearances represent 7 – 12-year troughs in 20 – 30-year WLP population cycles occurring synchronously at regional and perhaps continent-wide spatial scales as large as 50-500 million ha. This may represent the first documented case of natural population cyclicity in a Neotropical mammal. Because WLP populations often increase dramatically prior to a disappearance, we posit that their population cycles result from over-compensatory, density-dependent mortality. Our data also suggest that the increase phase of a WLP cycle is partly dependent on recolonization from proximal, unfragmented and undisturbed forests. This highlights the importance of very large, continuous natural areas that enable source-sink population dynamics and ensure re-colonization and local population persistence in time and space.
Order of Authors:	Jose M.V. Fragoso
	André P Antunes
	Mariana Altrichter
	Pedro AL Constantino
	Gala Zapata Rios
	Hani R El Bizri
	Richard E Bodmer
	Micaela Camino
	Benoit de Thoisy
	Robert B Wallace
	Thais Morcatty
	Pedro Mayor
	Cécile Richard Hansen
	Matthew T Hallett
	Rafael A Reyna-Hurtado
	Harald Beck

Powered by Editorial Manager® and ProduXion Manager® from Aries Systems Corporation

	Soledad de Bustos
	Alexine Keuroghlian
	Alessandra Nava
	Olga Montenegro
	Ennio Painkow Neto
	Kirsten M Silvius
**Additional Information:**	
**Question**	**Response**
**Financial Disclosure**<br><br>Enter a financial disclosure statement that describes the sources of funding for the work included in this submission. Review the submission guidelines for detailed requirements. View published research articles from *PLOS ONE* for specific examples.<br><br>This statement is required for submission and **will appear in the published article** if the submission is accepted. Please make sure it is accurate.<br><br>**Unfunded studies**<br>Enter: *The author(s) received no specific funding for this work.*<br><br>**Funded studies**<br>Enter a statement with the following details:<br>• Initials of the authors who received each award<br>• Grant numbers awarded to each author<br>• The full name of each funder<br>• URL of each funder website<br>• Did the sponsors or funders play any role in the study design, data collection and analysis, decision to publish, or preparation of the manuscript?<br>• **NO** - Include this sentence at the end of your statement: *The funders had no role in study design, data collection and analysis, decision to publish, or preparation of the manuscript.*<br>• **YES** - Specify the role(s) played.<br><br>* typeset	Authors were self funded for reanalyzing data already published
**Competing Interests**<br><br>Use the instructions below to enter a	The authors have declared that no competing interests exist

competing interest statement for this submission. On behalf of all authors, disclose any competing interests that could be perceived to bias this work—acknowledging all financial support and any other relevant financial or non-financial competing interests.<br><br>This statement is required for submission and **will appear in the published article** if the submission is accepted. Please make sure it is accurate and that any funding sources listed in your Funding Information later in the submission form are also declared in your Financial Disclosure statement.<br><br>View published research articles from *PLOS ONE* for specific examples.<br><br>**NO authors have competing interests**<br><br>Enter: *The authors have declared that no competing interests exist.*<br><br>**Authors with competing interests**<br><br>Enter competing interest details beginning with this statement:<br><br>*I have read the journal's policy and the authors of this manuscript have the following competing interests: [insert competing interests here]*<br><br>* typeset	
**Ethics Statement**<br><br>Enter an ethics statement for this submission. This statement is required if the study involved:<br><br>• Human participants<br>• Human specimens or tissue<br>• Vertebrate animals or cephalopods<br>• Vertebrate embryos or tissues<br>• Field research	N/A

Write "N/A" if the submission does not require an ethics statement.

General guidance is provided below. Consult the submission guidelines for detailed instructions. **Make sure that all information entered here is included in the Methods section of the manuscript.**

Format for specific study types

Human Subject Research (involving human participants and/or tissue)

- Give the name of the institutional review board or ethics committee that approved the study
- Include the approval number and/or a statement indicating approval of this research
- Indicate the form of consent obtained (written/oral) or the reason that consent was not obtained (e.g. the data were analyzed anonymously)

Animal Research (involving vertebrate animals, embryos or tissues)

- Provide the name of the Institutional Animal Care and Use Committee (IACUC) or other relevant ethics board that reviewed the study protocol, and indicate whether they approved this research or granted a formal waiver of ethical approval
- Include an approval number if one was obtained
- If the study involved *non-human primates*, add *additional details* about animal welfare and steps taken to ameliorate suffering
- If anesthesia, euthanasia, or any kind of animal sacrifice is part of the study, include briefly which substances and/or methods were applied

Field Research

Include the following details if this study involves the collection of plant, animal, or other materials from a natural setting:

- Field permit number
- Name of the institution or relevant body that granted permission

Powered by Editorial Manager® and ProduXion Manager® from Aries Systems Corporation

**Data Availability**<br><br>Authors are required to make all data underlying the findings described fully available, without restriction, and from the time of publication. PLOS allows rare exceptions to address legal and ethical concerns. See the PLOS Data Policy and FAQ for detailed information.<br><br>A Data Availability Statement describing where the data can be found is required at submission. Your answers to this question constitute the Data Availability Statement and **will be published in the article**, if accepted.<br><br>**Important:** Stating 'data available on request from the author' is not sufficient. If your data are only available upon request, select 'No' for the first question and explain your exceptional situation in the text box.<br><br>Do the authors confirm that all data underlying the findings described in their manuscript are fully available without restriction?	Yes - all data are fully available without restriction
**Describe where the data may be found in full sentences. If you are copying our sample text, replace any instances of XXX with the appropriate details.**<br><br>• If the data are **held or will be held in a public repository**, include URLs, accession numbers or DOIs. If this information will only be available after acceptance, indicate this by ticking the box below. For example: *All XXX files are available from the XXX database (accession number(s) XXX, XXX.*).<br>• If the data are all contained **within the manuscript and/or Supporting Information files**, enter the following: *All relevant data are within the manuscript and its Supporting Information files.*<br>• If neither of these applies but you are able to provide **details of access elsewhere**, with or without limitations, please do so. For example:	All data is included in the manuscript

Data cannot be shared publicly because of [XXX]. Data are available from the XXX Institutional Data Access / Ethics Committee (contact via XXX) for researchers who meet the criteria for access to confidential data.<br><br>*The data underlying the results presented in the study are available from (include the name of the third party and contact information or URL).*<br>• This text is appropriate if the data are owned by a third party and authors do not have permission to share the data.<br><br>* typeset	
Additional data availability information:	

Powered by Editorial Manager® and ProduXion Manager® from Aries Systems Corporation

Manuscript Click here to access/download;Manuscript;Fragoso et al 2021_draft.docx

Disappearances and population cycles of a large mammal in Neotropical forests

José M.V. Fragoso[1,2,3 *], André P. Antunes[4,5], Mariana Altrichter[6], Pedro A.L. Constantino[7], Galo Zapata-Ríos[8], Hani R. El Bizri[9,10], Richard E. Bodmer[11,12], Micaela Camino[13,14], Benoit de Thoisy[15], Robert B. Wallace[16], Thais Q Morcatty[17,18], Pedro Mayor[19,20,21], Cécile Richard-Hansen[22], Matthew T. Hallett[23,24,25], Rafael A. Reyna-Hurtado[26], Harald Beck[27], Soldedad de Bustos[28,29], Alexine Keuroghlian[30], Alessandra Nava[31], Olga L. Montenegro[32], Ennio Painkow Neto[33], Kirsten M. Silvius[34]

[1] Departamento de Zoologia, Universidade de Brasília, Brasília, DF, Brazil.

[2] Instituto Nacional de Pesquisas da Amazonia (Inpa/MCTIC) (INPA), Manaus, Brazil.

[3] California Academy of Sciences, San Francisco, California, United States of America.

[4] RedeFauna – Rede de Pesquisa em Diversidade, Conservação e Uso da Fauna da Amazônia, Tefé, Amazonas, Brazil.

[5] Instituto Nacional de Pesquisas da Amazônia (INPA), Manaus, Amazonas, Brazil.

[6] Co-chair, IUCN/SSC Peccary Specialist Group, Faculty Environmental Studies, Prescott College, Prescott, Arizona, United States of America.

[7] RedeFauna - Rede de Pesquisa em Diversidade, Conservação e Uso da Fauna da Amazônia - Tefé, Amazonas, Brazil.

[8] Wildlife Conservation Society – Ecuador Program. Quito, Ecuador.

[9] Department of Natural Sciences, Manchester Metropolitan University, Manchester, United Kingdom.

[10] Instituto de Desenvolvimento Sustentável Mamirauá, Tefé, Amazonas, Brazil

[11] Museum of Amazonian Cultures-Fundamazonia, Iquitos, Loreto, Perú.

[12] DICE, School of Anthropology & Conservation, University of Kent, Canterbury, United Kingdom.

[13] Proyecto Quimilero, Roosevelt 4344, CABA, Argentina.

[14] EDGE of Existence - Zoological Society of London, Regent's Park, London, England, United Kingdom.

[15] Kwata NGO, Cayenne; French Guiana

[16] Wildlife Conservation Society, Bronx, New York, United States of America.

[17] Department of Social Sciences, Oxford Brookes University, Oxford, United Kingdom.

[18] Instituto de Desenvolvimento Sustentável Mamirauá, Tefé, Amazonas, Brazil.

[19] Departament de Sanitat i d’Anatomia Animals, Facultat de Veterinària, Universitat Autònoma de Barcelona, Spain.

[20] Museo de Culturas Indígenas Amazónicas, Loreto, Iquitos, Peru.

[21] ComFauna, Comunidad de Manejo de Fauna Silvestre en la Amazonía y en Latinoamérica, Iquitos, Loreto, Peru.

[22] Office Français de la Biodiversité-DRAS/UPFSEO UMR EcoFoG. Juvignac, France.

[23] Department of Wildlife Ecology & Conservation, University of Florida, Gainesville, Florida, United States of America.

[24] Institute for the Environment & Sustainability, Miami University, Oxford, Ohio, United States of America.

[25] Center for International Forestry Research (CIFOR), Bogor, Indonesia.

[26] El Colegio de la Frontera Sur -Unidad Campeche: Campeche, Campeche, México.

[27] Co-Chair UCN/SSC Peccary Specialist Group. Department of Biological Sciences, Towson University, Towson, Baltimore, Maryland, United States of America.

[28] Secretaría de Ambiente y Desarrollo Sustentable de Salta, Santiago del Estero, Salta, Argentina,

[29] Fundación Biodiversidad Argentina, Suipacha, CABA, Argentina.

[30] Peccary Project/IUCN/SSC Peccary Specialist Group.

[31] Fiocruz ILMD Amazon, Adrianópolis, Manaus, Amazonas, Brazil.

[32] Instituto de Ciencias Naturales, Universidad Nacional de Colombia, Bogotá, Colombia.

[33] Tropical Sustainability Institute – TSI. Carapicuíba, São Paulo, Brazil.

[34] Department of Forest Resources and Environmental Conservation, Virginia Tech, Blacksburg, Virginia, United States of America.

* Corresponding author: José Manuel Vieira Fragoso (JMVF)

Emails: fragoso1@mac.com ; jose.fragoso@unb.br

Abstract

Many vertebrate species undergo population fluctuations that may be random or regularly cyclic in nature. Vertebrate population cycles in northern latitudes are driven by both endogenous and exogenous factors. Suggested causes of mysterious disappearances documented for populations of the Neotropical, herd-forming white-lipped peccary (*Tayassu pecari*, henceforth "WLP") include long distance or migratory movements, overhunting, extreme floods, or disease outbreaks. By analyzing 43 disappearance events across the Neotropics and 88 years of commercial and subsistence harvest data for the Amazon, we show that WLP disappearances are widespread and

occur regularly and at large spatiotemporal scales throughout the species' range. We present evidence that the disappearances represent 7 – 12-year troughs in 20 – 30-year WLP population cycles occurring synchronously at regional and perhaps continent-wide spatial scales as large as 50-500 million ha. This may represent the first documented case of natural population cyclicity in a Neotropical mammal. Because WLP populations often increase dramatically prior to a disappearance, we posit that their population cycles result from over-compensatory, density-dependent mortality. Our data also suggest that the increase phase of a WLP cycle is partly dependent on recolonization from proximal, unfragmented and undisturbed forests. This highlights the importance of very large, continuous natural areas that enable source-sink population dynamics and ensure re-colonization and local population persistence in time and space.

Introduction

Periodicity in animal population dynamics—i.e., population cycling—has long been observed in nature in northern vertebrate [small and medium sized mammals, birds, fish) and invertebrate populations [1, 2]. In general, cycling populations of vertebrates and insects show rapid decline phases and slow growth phases [2, 3]. Non-cyclic population dynamics also occur as random trends in population size, punctual, one-time decreases and increases, or irregular recurring increases and decreases with variable amplitude and periodicity.

The white-lipped peccary (*Tayassu pecari*, henceforth "WLP") is the only tropical forest ungulate that forms large, permanent, cohesive herds comprising hundreds of individuals [4]. Entire

populations have disappeared and eventually reappeared over large areas, with important consequences for forest composition and the livelihoods of forest-dependent peoples [4]. In the Amazon, the home range of a herd of about 400 animals may extend to 200 km^2 [4]. Considering an adult weight of 30 to 50 kg, such a herd represents 12,000 to 20,000 kg of biomass moving across the landscape, rooting up soils, consuming seeds, seedlings, plant parts and animal matter, dropping excreta, creating wallows, and providing abundant food for large cats, all processes that influence biotic communities [5] and the carbon cycle [6]. WLP population dynamics are therefore of interest in studies of forest ecology, as cyclicity in their abundance may permit periodic recruitment of plants and influence predator pressure on other prey species [7]. Fluctuations in WLP abundance also influence human ecology because, when abundant, WLPs are a very important source of protein for indigenous and rural people, representing up to 70 % of hunted biomass in some cases [4, 8]. While the WLP's unusual social organization and use of space is increasingly well-studied [9, 10], the repeatedly documented disappearance and reappearance of populations over large areas remain unstudied and poorly understood [4, 11].

Cycling can occur in mammal species with high reproductive rates when they are subject to non-linear or over-compensating density-dependent mortality. When mortality is linear and density-dependent, cycling can still occur if prolonged negative environmental or other conditions depress reproduction and / or extend high mortality rates into the decline phase of the cycle despite reduced densities [2, 3]. Although the conditions necessary for cycling to occur are understood and can be demonstrated mathematically, the specific drivers of cyclicity are diverse and vary within and among species. Endogenous factors include variations in reproductive output and other inherited qualities [12] or genetic drift [13], while exogenous factors include interactions

with predators [14], diseases [15], food availability [16, 17], and physical impacts, such as fires and floods [18]. Dispersal dynamics and social behaviors also influence the shape of cycles [2].

Potentially cyclic populations dynamics have been documented in small populations of temperate ungulates in fragmented ranges [19], but given the altered nature of these populations, it is difficult to assess whether these represent cases of cycling in the same way documented for snowshoe hares (*Lepus americanus*) and small mammals [14, 17], given the reduced size of the populations and the fragmented nature of their range.

In the 1980s, researchers in the Amazon began to report occasional local disappearances of WLP populations, attributing them to large-scale movements [20, 21], overhunting [22], or disease outbreaks [23]. Because WLPs are notoriously difficult to study in their remote, dense forest habitats, and due to their large home-range, we fill this gap in knowledge by compiling multiple sources of evidence on fluctuations in WLP abundance throughout the species' range and by analyzing the extent, timing and periodicity of documented disappearances. We also examine over-compensating density-dependent mortality as a possible explanation for the disappearances and recoveries in WLP populations.

Methods

Survey

We conducted a questionnaire-based survey of experts from 2016 to 2019. This questionnaire was sent to 58 Neotropical wildlife researchers, including all members of the IUCN/SSC Peccary Specialist Group. To complement the questionnaire, we conducted a Google Scholar search of

the key words white-lipped peccary, Tayassu *pecari* and peccary and extracted the same information as from survey respondents for instances of multiyear, extensive area disappearances. Independent reports from the same date and location were consolidated as one event, while separate reports from the same or adjacent locations within a year of each other were retained in the data set but counted as single events. Disappearances at sites separated by 100 km were considered distinct events. We estimated the mean years of absence, SD and IQR for cases at sites >100,000 ha that were surrounded by contiguous forest. In cases where a range of time was provided, we used the average of the range.

Commercial pelt trade and subsistence hunting record

We examined legal pelt trade records from 1932 to 1969 from 379 cargo manifests, port registries and financial documents [24], governmental statistics from Peru between 1946 and 2019 [25], and subsistence hunting records from 66 published and unpublished studies from 1965 to 2017 (S1) from the South-western Amazon. The combined dataset includes records of nearly 2 million WLPs hunted for their hides and meat over an estimated 686 million ha. We controlled for variation in hunting effort by calculating the proportion of WLP pelts and kills to other similarly preferred and priced species, the collared peccary (*Tayassu tajacu*). For each year and locality, we divided the number of WLP hunted by the total hunted of the two species (*WLP/WLP+CP)*. The resulting dataset contains 749 WLP hunting-proportion events for both commercial and subsistence purposes. We assume that the number of kills reflects the abundance of the species on the hunting grounds, and that when the number of hunted WLPs is low relative to CP, the WLP populations are low and individuals are difficult to find and kill. Conversely, if the proportion of WLP to CP is high, we assume that the WLP population is also high. Using the

proportion of hunted WLP to CP allowed the joint analysis of commercial and subsistence hunting data, by successfully controlling differences in hunting effort throughout time and space.

Population fluctuations analysis

We modeled the fluctuations of the WLP proportion through time as population fluctuations. The unknown proportion of the WLPs hunted in relation to CP in year t, for t = 1932 to 2019, denoted by $\gamma_i(t)$, is assumed to be a smooth curve over time. Since the proportion varied between 0 and 1 we used a beta inflated distribution model, $X(t) \sim BEINF(BI(t), \alpha, \gamma, \mu, \phi)$ – a mixture distribution between beta and Bernoulli distributions that allows values 0 and 1 for the response variable – where $BEINF(BI(t))$ denotes a beta inflated distribution with mean $BEINF(BI(t))$, μ location and ϕ scale parameters that control the probability density function, and two extra parameters α and γ, which model the probabilities at zero and one [26, 27]. To track the population fluctuation curve $\gamma_i(t)$ as a cubic spline [28, 29], we used gamlss package (Version 5.1-6) [30] in R language software [31], which estimates $\alpha, \gamma, \mu, \phi$ and spline parameters (number and position of knots for the curve $\gamma_i(t)$) by maximum likelihood with automatized Generalised Akaike Information Criteria for model selection [27]. We used the different Amazon river basins as a random factor due to different sample sizes among basins, and to possible non-independence of sampled localities within each basin.

Results and discussion

Literature review and expert reporting of disappearances

We recorded 67 reports from experts on 43 independent WLP disappearances at 38 sites in 9 countries (S1), occurring over a cumulative area of at least 50 million ha. Twenty-eight disappearance sites are in contiguous old-growth forest in the Amazon region, nine in forest fragments in Atlantic Forest in southern Brazil and northern Argentina, and one in rainforest in Guatemala (S1).

At small scales (<100,000 ha), we defined a disappearance as a sudden (no more than 12 months elapsed between the last documented normal to superabundant population level (see below) and a documented low or absent level) decline from previously documented normal to superabundance to low abundance or absence. In the absence of intensive searching, it is difficult to distinguish between low abundance and absence over larger areas; with the exception of the French Guiana case described below, such intensive searching has rarely been undertaken. We therefore defined a disappearance as occurring when no or very few WLP were sighted by frequent expert visitors to the area and / or local families and hunters for at least 3 years, and a reappearance as the first known documentation of normal population levels for an area after a period of absence. We describe five categories of abundance: zero, low, normal, high and superabundant. For example, a density of about 5 individuals per km^2 is low, and about 10 individuals per km^2 is normal in the Amazon [9, 32]. High densities equal about 15 individuals per km^2 whereas at superabundance densities can reach 20 to 100 or more individuals per km^2 [33]. Not all population declines had been documented continuously; in these cases, the absence periods were estimated by researchers. Large scale (>1,000,000 ha) disappearances may occur gradually over 2 to 5 years over a contiguous area, with the start of the event progressing outward from one location [34].

Two cases, one in Brazil and one in Guyana, illustrate rapid population crashes (S1). At Maracá Island Ecological Reserve in the northern Brazilian Amazon state of Roraima, WLPs were at a superabundant density (138.8 ind/km^2) in 1988 and herds were sighted almost every day during intensive searching [33]. WLPs then ceased to be detected in March 1989 and were not detected again despite continuous monitoring until a small herd of 30-40 individuals was seen in January 1991 and a second with 100-200 individuals was observed in September 1991 [4]. Herd size grew very slowly and by 2005 the population reached normal density for the Amazon, but still lower than the superabundant numbers just prior to the crash. In Guyana, intensive research over 3.5 years described a WLP population with high to superabundant densities of up to 25 ind./km^2 up until June 2010, when the study ended [35, 36]. WLP absence was documented starting in 2011 and for the next 7 years by camera trapping, transect surveys and hunter reports for the region (S1). As of 2019, WLP had not reappeared in most areas of Guyana.

Population absences or low abundances for 23 cases in contiguous forest lasted on average 10.3 years (SD = 4.9), with 50% lasting between 7 and 12 years. Some population lows were well documented through long-term field research. For example, in contiguous forest regions of the Amazon biome in French Guiana, WLPs were continuously monitored between 2000 and 2019. They were at easily detectable abundances throughout the territory in 2000 and 2001. Monitoring revealed a geographically expanding absence starting in 2003, reaching almost complete absence from 2006 to 2009 across the entire region. During 2012 and 2013, WLPs were again detected in several locations.

Longer absences are reported from discontinuous forests outside the Amazon. In the 41,704-ha Intervals State Park, Brazil, located in the largest remnant of Atlantic Forest fragment in São Paulo State, WLPs ceased to be detected in 1990 and were again detected in 2017 after a 27-year absence. In the larger Iguaçu National Park and adjacent northern Misiones province in Argentina, WLPs ceased to be detected around 1995. Years of surveys confirmed their absence until several individuals were detected with camera traps in 2016, likely immigrating from nearby forested areas in Argentina [37]. In Turvo State Park (Rio Grande do Sul State, Brazil), WLPs ceased to be detected in 1980 and have not been seen since then (S1).

At several sites, more than one disappearance event has been documented through time, pointing to population cyclicity. In and around Manu National Park, Peru, WLPs disappeared in 1978, reappeared 12 years later, then disappeared again in 2011. A few individuals were detected in 2015, but as of 2019, the population had not returned to pre-disappearance levels. Two distinct disappearance events occurred in the 275,531-ha Rio Jutaí Extractive Reserve, located in continuous, undisturbed forest in western Amazonas state, Brazil. Local inhabitants reported the start of the first disappearance between 1998-2002, with the timing depending on the location within the reserve. WLPs reappeared between 2006-2011, with the timing again depending on the site (S1). Researchers were present for the second disappearance, which began in 2013 near the mouth of the river (S1). By 2014 WLPs had also disappeared from the adjacent section of the watershed and in 2015 from the more distant headwater region. WLPs have not yet been detected at any location in the reserve. In Madidi National Park, Bolivia, WLPs disappeared in 1984 and were absent for 10 to 14 years, then reached pre-disappearance densities for several years, and disappeared again in mid-2017 along the Heath river, followed by the Tuichi and Hondo rivers in

2018 (S1). Also, in Amazonas state, Brazil, three independent disappearance events overlapping in time were reported in an area of more than 3 million ha including Amanã Sustainable Development Reserve, Unini River Extractive Reserve and Jaú National Park (S1).

Because researchers monitor a limited set of sites in a region, the true geographical extent of disappearances is usually not known. Nevertheless, we documented several cases of disappearances occurring at large spatial scales: 35% of the disappearances affected areas larger than 1-million ha (S1). In French Guiana, WLPs disappeared over the entire 8-million ha territory (Fig. 1). In Guyana, they disappeared over at least 5.4-million ha in the Rupununi region. In the northern Amazon of Brazil, WLPs disappeared over at least 4.8-million ha in Roraima state (Fig. 1). In Madidi National Park in Bolivia and Manu National Park in Peru, disappearances affected about 1-million and 1.7-million ha, respectively (S1).

Fig. 1 Locations of white-lipped peccary (*Tayassu pecari*) disappearances. Brown dots mark disappearances of small or unknown areal extent. Yellow lines mark the estimated extent for disappearances over larger regions. The 686 million ha western Amazon region, the source area for pelt and hunting data, is delineated in red. The blue line demarcates the Amazon biome. The disappearance site in Guatemala is not shown.

Long-term WLP population fluctuations in the western Amazon

We examined legal pelt trade records from 1932 to 2019 [24, 25] and subsistence hunting from 66 studies between 1965 and 2017 from the western Amazon. The combined dataset includes

records of almost 2 million WLPs hunted for their hides and meat over an estimated 686 million ha during 88 years (Fig.1). To detect fluctuations in WLP peccary returns while accounting for spatiotemporal variation in hunting effort, we relied on the relative frequency of hunted WLPs and collared peccaries (*Pecari tajacu,* CP). The two peccary species are among the most preferred species by subsistence hunters [35, 38] and prices for their skins were nearly identical during the period analysed [24], such that hunting effort is equal for the two species at any one time. By tracking the proportion of hunted WLP relative to total peccaries hunted of both species (WLP/WLP+CP) we control for the differences in overall hunting effort among years while assuming that hunting effort is equal for both species in any one year. Population fluctuations have not been observed or documented for CPs, which occur in small, territorial family groups (6 to 20 individuals) [39] rather than large, home-range-overlapping herds like WLPs. We modeled fluctuations in the WLP to CP proportion (WLP/WLP+CP) as a proxy for population fluctuation. Based on this time series (1932 to 2019), three synchronic growth and decline events in WLP abundance are evident over the western Amazon (Figs 1 and 2). Two large peaks (1941 and 2006) and one smaller peak (1978) were followed by rapid declines (late 1940s, and late 2000s), while a third peak (1975) showed a more gradual decline (perhaps due to limited data during this period). Increases were gradual in all cases—population growth required over 20 years to reach population maxima—whereas the steeper slopes of at least two declines evidence fast, large-scale population crashes occurring within about 5 years.

Fig. 2 Estimated multi-decadal population fluctuation of WLP based on the proportion of hunted WLP to the total of WLP plus hunted collared peccary (WLP/WLP+CP; 1932 to 2019) in the western Amazon. The shaded gray area indicates 95% confidence interval.

Circles are individual data points. Data from commercial pelt and subsistence hunting for meat.

The time series of WLP hunting harvests in the western Amazon analyzed here provide strong empirical evidence of population cycling in a large tropical vertebrate over large geographical areas. WLP population cycles appear to last 20 to 30 years from peak to peak, with a rapid population decline phase over 1 to 5 years, an absence or low-abundance phase of 7 to 12 years, followed by a slow growth phase of about 20 years.

Enigmatic causes of WLP disappearances: is it a cyclic species?

The enigmatic population dynamics pattern described here is unexpected for any large forest mammal and previously undocumented for any Neotropical mammal. The compiled data from multiple sources on timing and spatial extent of WLP disappearance suggests that the dynamics are periodic (and therefore cyclical), and occur at regional scales of about 1 - 500 million ha. Such periodicity could be driven by an external, periodic driver, or by intrinsic, density-dependent factors. In the past, several external factors have been suggested. We review those here, but note that none of them can account for periodic, cyclic fluctuations. We also propose intrinsic, density-dependent dynamics as an alternative explanation, while noting that some of the external factors could contribute to extended populations lows and, in the case of diseases, may be part and parcel of the density-dependent dynamics.

Next, we discuss whether any of the drivers previously suggested could account for such a widespread phenomenon.

Hunting

Although WLP meat is preferred by local peoples and the species is vulnerable to overhunting [40], many studies have found WLP hunting to be sustainable [35, 36, 41]. It is unlikely that the WLP disappearances we documented are caused by hunting, because 1) most disappearances occurred in unpopulated, remote areas of contiguous forest; and 2) there is no reason for or indication of historic changes in hunting effort sufficient to drive the boom and crash periods. For instance, in Acre State, Brazil and Loreto Department, Peru, number of hunters remained relatively constant in the monitored sites during the 2000s and 2010s. Continued reduced survival due to hunting can however amplify natural population declines [4]. Hunting should be more deleterious in small, isolated forest remnants and less so in the continuous Amazon forest with undisturbed source areas that buffer impacts of local hunting on WLP populations [4, 11].

Local and regional movements driven by environmental stressors

WLP disappearances have also been attributed to long distance and / or migratory events related to environmental stressors [4, 21]. In the western Amazon WLPs have been observed to move out of flooded forest to non-flooded tierra-firme forest during high floods, sometimes crossing large rivers [18]. In cases of extreme flooding, mortality may be occurring together with these movements. For example, WLPs monitored intermittently through line-transects, hunter interviews and camera trap methods since 1990 in the Yavari-Samiria region an area covering 4 million ha of terra-firme and flooded forests in the Peruvian Amazon [18, 42] move seasonally from flooded to upland forests, and population fluctuations in each of these habitats may be related to intensity of flooding. In the tierra-firme forests of the Yavari valley WLPs have shown

extreme population fluctuations over 30 years, including a decline from a high of 15 ind./km^2 in 200 to 2.0 ind./km^2 in 2004 and a complete disappearance in 2013. In contrast, in flooded forests the WLPs showed less extreme oscillations of 7.7 ind./km^2 +- 3.4 (SD), but also disappeared between 2013 and 2017 following historically high and prolonged floods that began in 2011 and lasted through 2015. This regional disappearance following extreme flooding was reflected in meat sales in the Iquitos bush meat market, which provides a measure of WLP harvest from the Loreto Region, an area of 38 million ha that includes the Yavari landscape [43]. In 1996 there were 4,510 individuals sold, in 2006 3,170 were sold, and then a dramatic decrease was seen in 2013 when only 253 WLP were sold. In 2018 sales rebounded with 3,227 individuals sold.

The bearded pig (*Sus barbatus*) of Southeast Asia periodically forms large herds that then move long distances, presumably tracking fruiting by preferred trees [44]. Our knowledge on WLP movements is still too incipient to infer WLP disappearances from food-related migratory movements, especially across the very large spatial scales observed.

Disease

The gregarious nature of WLP, their unusually large herd size for a forest dwelling ungulate species, and their overlapping home ranges that allow herds to use that same feeding sites and wallows may set the conditions for rapid disease spread that locally eliminates multiple herds and entire populations [45]. Emmons [23] proposed that a local disease outbreak caused a WLP population disappearance event in Manu National Park, Peru. In 1997, Fragoso [4, 11] postulated epidemic outbreaks at regional scales after noting that the northern Roraima, Brazil disappearance event was concurrent with a foot and mouth disease outbreak in livestock in adjacent ranchlands.

He reported that epidemics could cause disappearances at large scales (millions of ha) [4], as has been documented for temperate and tropical Old-World ungulates [46, 47]. He also proposed that disease outbreaks could be an intrinsic and recurrent consequence of the species' unique social organization, herd size and movements. WLP carcasses untouched by predators or humans were found in northern Roraima during this period. Similarly, WLP carcasses were found during unexplained disappearances in Manu and Tambopata-Peru, and Guatemala. Diseased but live individuals were also sighted at Viruá National Park- Brazil and Mexico. In all other events (n=30) however, no carcasses were reported and no causes for the disappearances were proposed. Novel pathogen(s) infecting WLP have not been identified and it is still an open question whether disease outbreaks could drive population collapses, under their own synchrony or in synergy with other stressors.

Density dependent overcompensation

An alternative explanation for the WLP disappearances is an over-compensation in density-dependent responses, with the most likely proximate causes being decreased reproductive rates and higher mortality caused by weakened condition in super-abundant populations. The gregarious nature of WLP, whose herds reach up to some hundreds of individuals in their population maxima, as well as the relatively species-specific high reproductive rate (1.64-1.77 young per litter, 158 days of pregnancy, and 18 months age of first reproduction) [48, 49, 50] create plausible conditions for exceeding carrying capacity, subsequently triggering over-compensating mortality [19] and negative maternal effects [3], which could explain the rapid crash of WLP populations we report. Thus, limited food, diseases, parasites or any hidden physiological stressor would be amplified by high densities resulting in rapid crash periods for a

species whose population cyclicity is mostly driven by density-dependent abundance and growth. To our knowledge population cycles have not been documented, studied or confirmed for any Neotropical mammal, and non-cyclic population fluctuations, while occasionally observed [44, 51], have not been documented or studied.

The observation that declines are often preceded by super abundance concurs with a density-dependent explanation. When WLP populations reach high numbers, food becomes scarce, female reproductive productivity decreases, and there is greater infant and adult mortality. The population goes into a fast retraction leading to declines and disappearances. External drivers, including a decline in food or habitat quality independent of population increase, are not necessary explanations in this scenario, but could play a role by depressing population recovery, as in the case of disease affecting weakened animals or habitat reduction with crowding in the remaining habitat due to flooding.

Anthropological studies support the hypothesis of cyclicity in WLP populations. The WLP is highly prized as food by indigenous peoples [35] and plays a prominent role in their religious beliefs [52-54]. Although our own data are restricted to recent history, WLP disappearances appear to have occurred for long enough to be incorporated into the cosmologies and beliefs of various indigenous peoples in the Amazon [4, 52-54], which suggests that severe population fluctuations are a characteristic of the species. Several ethnic groups describe disappearance events as linked to the death of a powerful shaman; their return can only be secured by another shaman using special cultural activities [4, 54].

Re-appearances and conservation measures

WLP re-appearance consisting of gradually increasing numbers of individuals may result from population growth from a few remaining individuals or re-colonization [4]. Their herding social structure may mean that populations cannot reach high growth rates until herds reach a threshold size; this, together with the need for immigration from other populations to re-establish local populations may be factors that delay recovery. The period of absence or low abundance for WLP in continuous habitat is around 7-12 years at the large local scale and 20 years in the large regional scale. On the other hand, the absence period was over 20 years in sites with high forest fragmentation due to anthropogenic impacts, suggesting that in the absence of dispersal, regular cycling dynamics are interrupted and a population may go extinct.

Immigration is considered a factor in the growth phase of other species cycling populations [2]. If immigration is important in WLPs, then this suggests that there should be a lag time in the recovery of adjacent populations. For example, when the WLPs crashed in the Brazilian Yanomami Indigenous territory (Amazon River drainage area [4]; the Yanomami reported that WLPs started returning from over the Parima mountains from Venezuela (Orinoco River drainage area). This implies that the population in Venezuela was in a growth phase, which was driving outmigration of the animals. While we documented cycling as occurring throughout the Amazon basin, our data indicates that synchronicity in cycling occurs at the local or meso-scale rather than at the basin-wide scale. This would create a source-sink dynamic across areas with dispersal connecting populations across the entire basin.

The need for dispersal and repopulation events indicates the importance of large continuous areas for the maintenance of a WLP metapopulation and source-sink dynamics [55]. The large oscillations in WLP populations make them vulnerable to local extinctions and conservation actions are needed when the populations are low. Loss of WLP populations alters religious practices [54] and may induce human emigration [36]. It also contributes to the worldwide collapse of large mammals [5] and global defaunation [56].

Acknowledgments

J.M.V.F. thanks the Yanomami people for documenting the return of WLP to their area in the 1990s, and the Makushi and Wapichan people for tracking their hunting returns so precisely. Thanks also to Roan McNab, Luis Pacheco, Arnaud Desbiez, Mineração Rio do Norte and the Biota Projetos e Consultoria Ambiental for providing information. Thanks to Bill Magnusson and Albertina Lima for providing a home at INPA for the writing of a first draft of the manuscript.

References

1. Krebs CJ. Population cycles revisited. J. Mammal. 1996;**77**: 8-24.

2. Myers JH. Population cycles: generalities, exceptions and remaining mysteries. Proc. R. Soc. Lond. B. 2018;285: 20172841.

3. Ginzburg L, Colyvan M. Ecological Orbits: How planets move and populations Grow. New York: Oxford University Press; 2004.

4. Fragoso JMV. A long-term study of white-lipped peccary (*Tayassu pecari*) population fluctuation in northern Amazonia. In: Silvius K, Bodmer RE, Fragoso JMV, editors. People in

Nature. New York: Wildlife Conservation in South and Central America; Columbia University Press, 2004. pp. 286-296.

5. Ripple WJ. Newsome TM, Wolf C, Dirzo R, Everatt KT, Galetti M, et al., Collapse of the world's largest herbivores. Science Adv. 2015;1: e1400103.

6. Sobral M, Silvius MK, Overman H, Oliveira LF, Raab TK, Fragoso J. Mammal diversity influences the carbon cycle through trophic interactions in the Amazon. Nat. Ecol. Evol. 2017; 1: 1670-1676.

7. Fragoso JMV. The role of trophic interactions in community initiation, maintenance and degradation**.** In: Burslem D, Pinard M, Hartley S. editors. Biotic Interactions in the Tropics: their role in the maintenance of species diversity. England: Cambridge University Press; 2005. pp. 310-327.

8. Constantino PAL, Fortini LB, Kaxinawa FRS, Kaxinawa AM, Kaxinawa ES, Kaxinawa AB, et al. Indigenous collaborative research for wildlife management in Amazonia: the case of Kaxinawá, Acre, Brazil. Biol. Conserv. 2008;141: 2718-2729.

9. Richard-Hansen C, Berzins R, Petit M, Rux O, Goguillon B, Clément L. Movements of White-Lipped Peccary in French Guiana. In: Reyna-Hurtado R, Chapman CA, editors. Movement Ecology of Neotropical Forest Mammals: Focus on Social Animals. Cham: Springer; 2019. pp. 57-75.

10. Reyna- Hurtado R, Beck H, Altrichter M, Chapman CA, Bonnell TR, Keuroghlian A, et al. What ecological and anthropogenic factors affect group size in White- lipped Peccaries (*Tayassu pecari*)? Biotropica. 2016; 48: 246-254.

11. Fragoso JMV. Desapariciones locales del baquiro labiado (*Tayassu pecari*) en la Amazonía: migración, sobre-cosecha o epidemia. In: Fang T, Bodmer R, Aquino R, Valqui M, editors.

Manejo de Fauna Silvestre en la Amazonía. La Paz, Bolivia: United Nations Development Program-Global Environmental Facility, Universidad de Florida, Instituto de Ecología. 1997. pp 309-312.

12. Ginzburg LR, Taneyhill DE. Population cycles of forest Lepidoptera: a maternal effect hypothesis. J Anim Ecol. 1994;63: 79-92.

13. Donohue K. Completing the cycle: maternal effects as the missing link in plant life histories. Phil. Trans. Royal Soc. B: Biol. Sci. 2009; 364**:** 1059-1074.

14. Krebs CJ, Boutin S, Boonstra R, Sinclair ARE, Smith JNM, Dale MRT, et al. Impact of food and predation on the snowshoe hare cycle. Science. 1995; 269: 1112–1115.

15. Anderson RM, May RM. Infectious diseases and population cycles of forest insects. Science. 1980;210: 658-661.

16. Turchin P, Batzli GO. Availability of food and the population dynamics of arvicoline rodents. Ecology. 2001;82**:** 1521-1534.

17. Oli MK, Krebs C J, Kenney AJ, Boonstra R, Boutin S, Hines JE. Demography of snowshoe hare population cycles. Ecology. 2020;101: e02969.

18. Bodmer R, Mayor P, Antunez M, Chota K, Fang T, Puertas P, et al. Major shifts in Amazon wildlife populations from recent intensification of floods and drought. Cons. Biol. 2018;32: 333-344.

19. Clutton-Brock TH, Illius AW, Wilson K, Grenfell BT, MacColl ADC, Albon SD. Stability and instability in ungulate populations: an empirical analysis. Am. Nat. 1997;1: 195-219.

20. Kiltie RA. Distribution of palm fruits on a rain forest floor: why white-lipped peccaries forage near objects. Biotropica. 1981;14: 141-145.

21. Bodmer RE. Responses of ungulates to seasonal inundations in the Amazon floodplain. J. Trop. Ecol. 1990;6: 191-201.

22. Vickers WT. Hunting yields and game composition over ten years in an Amazon Indian territory. In: Robinson JG, Redford KH, editors. Neotropical Wildlife Use and Conservation**.** Chicago: University of Chicago Press; 1991. pp. 53-81.

23. Emmons LH. Comparative feeding ecology of felids in a neotropical rainforest. Behav. Ecol. Sociobiol. 1987;20: 271-283.

24. Antunes AP, Fewster RM, Venticinque EM, Peres CA, Levi T, Rohe F, et al. Empty forest or empty rivers? A century of commercial hunting in Amazonia. Sci. Adv. 2016; 2: e1600936.

25. Ministerio del Ambiente MINAM. Dictamen de Extracción No Perjudicial (DENP) de los cueros de pecaríes (Pecari tajacu y Tayassu pecari) en el Perú. Ministerio del Ambiente, Dirección General de Diversidad Biológica. Lima, Perú. 2020. Available from: https://www.gob.pe/minam#publicaciones.

26. Ospina R, Ferrari SL. Inflated beta distributions. Stat. Papers**.** 2010;51: 111.

27. Stasinopoulos MD, Rigby RA, Heller GZ, Voudouris V, De Bastiani F. Flexible Regression and Smoothing: using GAMLSS in R. 1st edition. Boca Raton: CRC Press; 2017.

28. Hastie TJ, Tibshirani RJ. Generalized Additive Models (Chapman & Hall/CRC Monographs on Statistics and Applied Probability Book 43). 1st edition. Boca Raton: CRC press, 1990.

29. Fewster RM, Buckland ST, Siriwardena GM, Baillie SR, Wilson JD. Analysis of population trends for farmland birds using generalized additive models. Ecology. 2000;81: 1970-1984.

30. Rigby RA. Stasinopoulos DM. Generalized additive models for location, scale and shape. J R Stat. Soc. Ser. C Appl. Stat. 2007;54: 507-554.

31. Dalgaard P. (Producer) R Development Core Team. R: A language and environment for statistical computing. 2010. Computer program, http://www.R-project.org/

32. Fragoso JMV. Home range and movement patterns of white-lipped peccary (*Tayassu pecari*) herds in the northern Brazilian Amazon. Biotropica. 1998;30: 458-469.

33. Fragoso JMV. White-lipped peccaries and palms on the Ilha de Maracá. In: Milliken W, Ratter JA, editors. Maracá: The Biodiversity and Environment of an Amazonian Rainforest. New York: Wiley & Sons; 1998. pp. 15-64.

34. Richard-Hansen C, Surugue N, Khazraie K, Le Noc M, Grenand P. Long-term fluctuations of white-lipped peccary populations in French Guiana. Mammalia. 2014;78: 291-301.

35. Fragoso JMV, Levi T, Oliveira LFB, Luzar JB, Overman H, Read JM et al. Line transect surveys under detect terrestrial mammals: implications for the sustainability of subsistence hunting. PLoS ONE. 2016;1: e0152659.

36. Iwamura T, Lambin EF, Silvius KM, Luzar JB, Fragoso JMV. Agent-based modeling of hunting and subsistence agriculture on indigenous lands: understanding interactions between social and ecological systems. Environ. Modell. & Softw. 2014;58: 109-127.

37. Brocardo CR, da Silva MX, Delgado LE, Galetti M., White-lipped peccaries are recorded at Iguaçu National Park after 20 years. Mammalia**,** 2017; 81**:** 519-522.

38. Constantino PAL. Deforestation and hunting effects on wildlife across Amazonian indigenous lands. Ecol. Soc. 2016;21**:** 1-10.

39. Fragoso JMV. Perception of scale and resource partitioning by peccaries: behavioral causes and ecological implications. J. Mammal 1999;80**:** 993-1003.

40. Altrichter M, Taber A, Beck H, Reyna-Hurtado R, Lizarraga L., Keuroghlian A, et al. Range-wide declines of a key Neotropical ecosystem architect, the Near Threatened white-lipped peccary *Tayassu pecari*. Oryx. 2012;46: 87-98.

41. Robinson JG, Bodmer RE. Towards wildlife management in tropical forests. J. Wildl. Manag. 1999; 63: 1-13.

42. Bodmer RE. Managing Amazonian wildlife: biological correlates of game choice by detribalized hunters. Ecol. Appl. 1995;5: 872-877.

43. Mayor P, El Bizri HR, Morcatty TQ, Moya K, Bendayán N, Solis S, et al. Wild meat trade over the last 45 years in the Peruvian Amazon. Cons. Biol. (Forthcoming). doi:10.1111/cobi.13801.

44. Caldecott JO, Blouch RA, Macdonald AA. The bearded pig (*Sus barbatus*). In: Oliver WLR, editor. Pigs, Peccaries, and Hippos: Status Survey and Conservation Action Plan. Gland, Switzerland: IUCN/SSC Pigs and Peccaries Specialist Group, IUCN/SSC Hippo Specialist Group; 1993. pp 136-145.

45. Weaver SC, Reisen WK. Present and future arboviral threats. Antiviral Research**.** 2010;85: 328-345.

46. Young TP. Natural die-offs of large mammals: Implications for conservation. Conserv. Biol. 1994; 8: 410-418.

47. Smith KF, Sax DF, Lafferty KD. Evidence for the role of infectious disease in species extinction and endangerment. Conserv. Biol. 2006;20: 1349-1357.

48. Eisenberg JF, Redford KH. The contemporary mammalian fauna. Mammals of the Neotropics-the central Neotropics, 3. 1st edition. Chicago; University of Chicago Press; 1999.

49. Mayor PG, Bodmer RE, Bejar ML. Reproductive performance of the wild collared peccary (*Tayassu tajacu*) female in the Peruvian Amazon. Eur. J. Wildl. Res. 2010;56**:** 681-684.

50. Mayor PG, El Bizri H, Bodmer RE, Bowler M. Assessment of mammal reproduction for hunting sustainability through community- based sampling of species in the wild. Conserv. Biol. 2017;31**:** 912-923.

51. Bodmer RE, Mayor P, Antunez M, Fang T, Chota K, Yuyarima TA, et al. Wild meat species, climate change, and indigenous Amazonians. J. Ethnobiol. 2020;40: 218-233.

52. Donkin RA. The peccary: with observations on the introduction of pigs to the New World. Trans. Am. Philos. Soc. 1985;75: 1-152.

53. Fausto C. Feasting on people: eating animals and humans in Amazonia. Curr. Anthropol. 2007; 48: 497-530.

54. Virtanen PK. The death of the chief of peccaries: The Apurinã and the scarcity of forest resources in Brazilian Amazonia. In: Reyes-Garcia V, Pyhälä A, editors. Hunter-gatherers in a Changing World. 1st edition. Cham: Springer; 2017. pp. 91-105.

55. Pulliam HR. Sources, sinks, and population regulation. Am. Nat. 1988; 132: 652-661.

56. Dirzo R. Young HS, Galetti M, Ceballos G, Isaac NJB, Collen B. Defaunation in the Anthropocene. Science. 2014;345: 401-406.

Supporting information

S1 File. Reports from experts on 43 independent WLP disappearances at 38 sites in 9 countries.

Click here to access/download;Figure;Fig1.tiff.tif

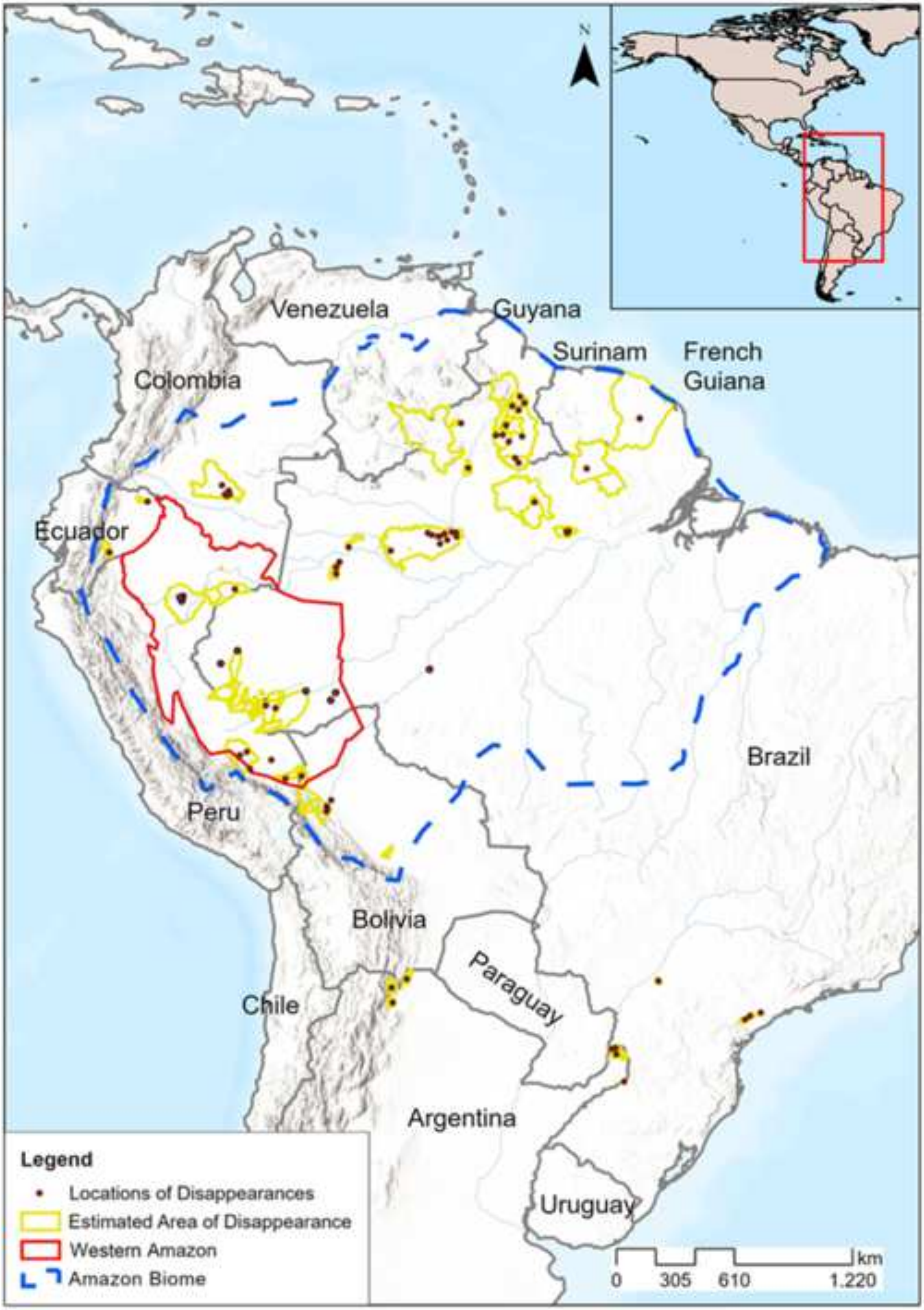

Click here to access/download;Figure;Fig2.tiff

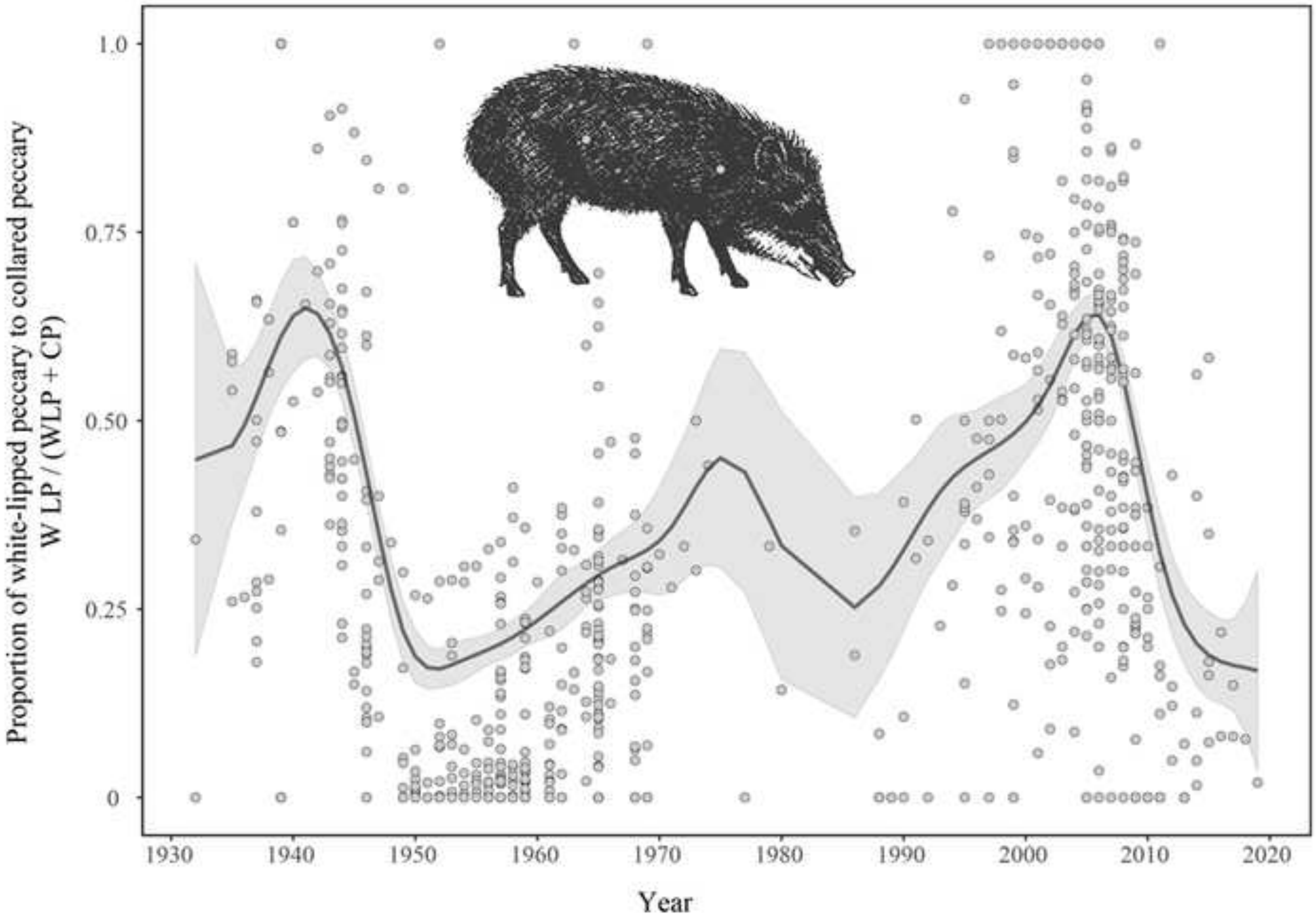

Click here to access/download
Supporting Information
S1_file.docx
