# Supplementary material for: Large-scale population disappearances and cycling in the white-lipped peccary, a tropical forest mammal
Source: PLoS One. 2022 Oct 20;17(10):e0276297. doi: 10.1371/journal.pone.0276297 (PMC9584423; doi:10.1371/journal.pone.0276297)
Supplement: Attachment — Submitted filename: Response to Reviewers.docx [file pone.0276297.s004.docx]

**Response to Reviewers**

Bilal Habib
Academic Editor
PLOS ONE

July 16, 2022

Dear Bilal,

After responding to the reviewers’ and editor’s comments, please accept our resubmission of the manuscript “Disappearances and population cycles of a large mammal in Neotropical forests” (PONE-D-21-25351), now titled “**Large-scale population disappearances and cycling in the White-lipped peccary, a tropical forest mammal”.** Comments and responses are as follows:

**Responses to editor:**

2. Please include additional information regarding the survey or questionnaire used in the study and ensure that you have provided sufficient details that others could replicate the analyses. For instance, if you developed a questionnaire as part of this study and it is not under a copyright more restrictive than CC-BY, please include a copy, in both the original language and English, as Supporting Information.

Response: The questionnaire we applied has been added as supplemental material, labelled Supplemental S1 Table

3. We note that the grant information you provided in the ‘Funding Information’ and ‘Financial Disclosure’ sections do not match. When you resubmit, please ensure that you provide the correct grant numbers for the awards you received for your study in the ‘Funding Information’ section.

Response: None of the authors are reporting funding support for this study.

4. In your Data Availability statement, you have not specified where the minimal data set underlying the results described in your manuscript can be found. PLOS defines a study's minimal data set as the underlying data used to reach the conclusions drawn in the manuscript and any additional data required to replicate the reported study findings in their entirety. All PLOS journals require that the minimal data set be made fully available. For more information about our data policy, please see <http://journals.plos.org/plosone/s/data-availability>.

Response: All data used in the study are already included in the tables and figures. There are no additional data sets to be referenced.

5. We note that Figure 1 in your submission contain map images which may be copyrighted. All PLOS content is published under the Creative Commons Attribution License (CC BY 4.0), which means that the manuscript, images, and Supporting Information files will be freely available online, and any third party is permitted to access, download, copy, distribute, and use these materials in any way, even commercially, with proper attribution. For these reasons, we cannot publish previously copyrighted maps or satellite images created using proprietary data, such as Google software (Google Maps, Street View, and Earth). For more information, see our copyright guidelines: <http://journals.plos.org/plosone/s/licenses-and-copyright>.

Response: The map in Figure 1 is not proprietary information nor is it copyrighted.

**Response to Reviewer**

1. The title should specifically mention the studied species rather using “a large mammal” for more clarity on the manuscript.

Response: We have changed the title to reference the species, as follows: Large-scale population disappearances and cycling in the White-lipped peccary, a tropical forest mammal

2. It will be better if authors will keep the unit of areas uniform in the entire study (Km2 or Hectare)

Response: We changed to Km^2^ in the tables but use hectares throughout the manuscript text.

3. The site specific information on genetic make-up or inbreeding have not been discussed as a factor of disappearance of the population in isolated patches. Authors should take a note on this and incorporate with supporting information in the manuscript.

Response: The reviewer suggested we discuss the existence, or not, of genetic factors that could affect disappearances. We do not understand this suggestion, as we do not believe there are genetic drivers of population cycling in WLP. Therefore, we have not added text to address the suggestion. We have already noted that population declines in forest fragments may become long term disappearance or local extinctions, due to limited resources and lack of connectivity to source areas

4. The cumulative impacts of different aspects such hunting, environmental stressors, disease in population cycle should be analyzed with existing dataset and can be discussed. At this state authors are touching upon the different aspects but a cumulative impact on population fluctuation is missing.

Response: There were already comments in appropriate sections in the manuscript on potential cumulative impacts, but we have also added a dedicated section on potential cumulative impacts on page 20.

5. Authors should provide more information emphasizing on the factors for the recolonization of the species.

Response: This is beyond the scope of this study, as the existence of recolonization, and the form it takes, likely varies from site to site and has rarely been documented. We have added a comment concerning this under the section Reappearances on page 21

**Please note** that additional comments made by the reviewer in the pdf version of the manuscript have been addressed in the manuscript and our responses can be viewed in the Word Track Changes copy.
